# Comparative Analysis of Multiple Neurodegenerative Diseases Based on Advanced Epigenetic Aging Brain

**DOI:** 10.3389/fgene.2021.657636

**Published:** 2021-05-20

**Authors:** Feitong Shi, Yudan He, Yao Chen, Xinman Yin, Xianzheng Sha, Yin Wang

**Affiliations:** ^1^Department of Biomedical Engineering, School of Fundamental Sciences, China Medical University, Shenyang, China; ^2^Tumor Etiology and Screening Department of Cancer Institute and General Surgery, The First Affiliated Hospital of China Medical University, Shenyang, China

**Keywords:** aging, neurodegenerative disease, cellular homeostasis, network analysis, supervised machine learning

## Abstract

**Background:** Neurodegenerative Diseases (NDs) are age-dependent and include Alzheimer’s disease (AD), Parkinson’s disease (PD), progressive supranuclear palsy (PSP), frontotemporal dementia (FTD), and so on. There have been numerous studies showing that accelerated aging is closely related (even the driver of) ND, thus promoting imbalances in cellular homeostasis. However, the mechanisms of how different ND types are related/triggered by advanced aging are still unclear. Therefore, there is an urgent need to explore the potential markers/mechanisms of different ND types based on aging acceleration at a system level.

**Methods:** AD, PD, PSP, FTD, and aging markers were identified by supervised machine learning methods. The aging acceleration differential networks were constructed based on the aging score. Both the enrichment analysis and sensitivity analysis were carried out to investigate both common and specific mechanisms among different ND types in the context of aging acceleration.

**Results:** The extracellular fluid, cellular metabolisms, and inflammatory response were identified as the common driving factors of cellular homeostasis imbalances during the accelerated aging process. In addition, Ca ion imbalance, abnormal protein depositions, DNA damage, and cytoplasmic DNA in macrophages were also revealed to be special mechanisms that further promote AD, PD, PSP, and FTD, respectively.

**Conclusion:** The accelerated epigenetic aging mechanisms of different ND types were integrated and compared through our computational pipeline.

## Introduction

With the further extension of human life, the number of elderly people is increasing and the incidence rate of senile neurodegenerative diseases (ND) is also rising (Kovacs, 2017). Although human life expectancy has been improved in recent years, ND have become the most common diseases affecting elderly populations (Heemels, 2016), with a large number of people being affected by Alzheimer’s disease (AD), Parkinson’s disease (PD), frontotemporal dementia (FTD), and progressive Supranuclear Palsy (PSP). Life is disrupted by the development of NDs in aging patients, with concomitant implications in terms of social resources and economic costs. It is estimated that the prevalence of NDs will increase to 131 million in the next few decades (Santiago et al., 2020), meaning that NDs have become an important topic of global concern.

A large amount of epidemiological evidence has shown that NDs are closely related to advanced brain aging, which is often considered to be one of the driving factors of ND (Mayne et al., 2020). There were a series of risk factors that interact with each other in the aging brain coordinately, where the immune system is dysregulated in abnormal aging (Liang et al., 2017). For example, chronic inflammation was considered to be one of the pathogenic factors of NDs in elderly people (Liang et al., 2017). In addition, the functions in the mitochondrial OXPHOS system are diminished in ND patients (Grimm and Eckert, 2017). Moreover, the accumulation of mitochondrial dysfunction (such as mtDNA mutation, increased oxidative stress, and mitochondrial transport/distribution defects) are more serious in ND (Grimm and Eckert, 2017).

**FIGURE 1:**
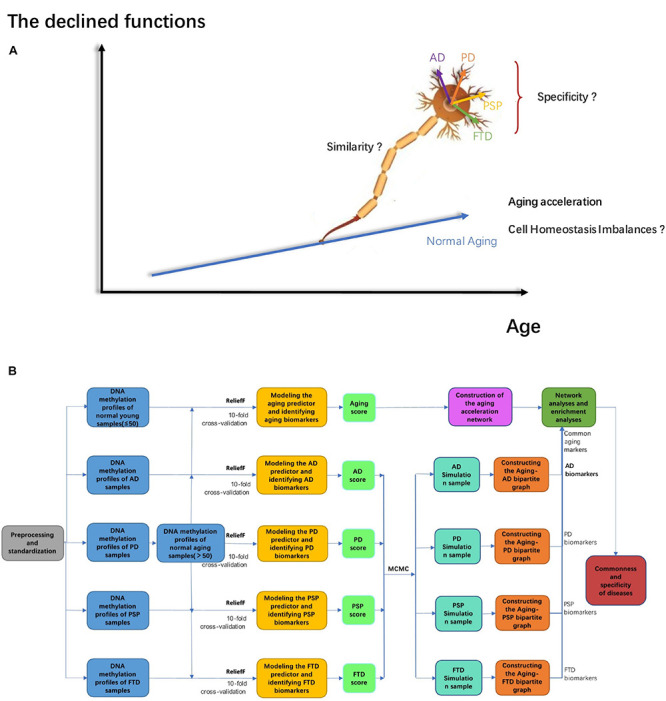
**(A)** The mechanism hypothesis diagram of ND; **(B)** the workflow in our work.

There are a series of common characteristics of the advanced aging brain shared by different ND types, such as imbalances in cellular homeostasis and advanced cell death (Xu et al., 2020). If the cells cannot return to homeostasis for a long time, it may lead to irreversible abnormal cell death, along with continuous dysfunctions or the pathological state (Xu et al., 2020). Cellular homeostasis is thought to be related to cell cycle arrest, cell senescence, apoptosis, and autophagy (Dodig et al., 2019). The DNA damage repair response, imbalance of cellular energy metabolisms, and immune homeostasis can lead to cellular homeostasis disorder. For instance, the protein stabilization network is particularly important in neurons, and its abnormality was considered to be closely related to ND. In addition, the nervous system diseases shared common pathogenic factors, such as oxidative stress, environmental stress, and protein dysfunction, disrupting the protein stability in cells. Studies have proven that the mechanisms related to protein stability might be the basis of the etiology of ND (Höhn et al., 2020). Furthermore, there are other pathogenic factors shared by different ND types in the context of aging brains, such as oxidative stress, extracellular fluid, and protein dysfunction, which are also related to imbalances in cellular homeostasis. For example, research has proved that the mechanisms relating to protein stability might be the basis of the etiology of NDs (Höhn et al., 2020). Various studies have also reported that metal ion homeostasis (e.g., copper, iron, and zinc) is dysregulated during advanced brain aging, then NDs are induced. The excessive accumulation of metal ions has been found in a large number of ND patients (Ashraf et al., 2018). In short, the mechanism of ion imbalance in neurons is also thought to be related to NDs (Jomova et al., 2010). Thus, both ion homeostasis disorder and protein homeostasis disorder led to irreversible cellular homeostasis disorders. Based on previous reports, we can hypothesize that cellular homeostasis imbalance is one of the most important risk factors of ND during advanced brain aging (Figure 1A).

Although a series of studies have shown that the occurrence of ND was mainly due to aging acceleration as well as the imbalance of cellular homeostasis, resulting in cell dysfunction and pathological state (Bohlen et al., 2019), the relative mechanisms still need to be explored more thoroughly. Machine learning technology can be leveraged to classify healthy and diseased populations (Camacho et al., 2018). Moreover, using a prediction model based on omic profiles to identify potential biomarkers is informative to the experimental design, evaluation, diagnosis, and treatment of ND (Camacho et al., 2018). There are also some studies using network methods to analyze the common and specific molecular characteristics of different ND types, but the mechanisms of how ND was related/triggered by accelerated aging as well as cellular homeostasis imbalances, still need to be investigated more systematically (Stopa et al., 2018; Santiago et al., 2020). We therefore urgently require explorations of these mechanisms based on proper datasets.

In this paper, the relationship between aging, cellular homeostasis, and ND (including AD, PD, PSP, and FTD), as well as the relative mechanisms involved, were analyzed by our computational pipeline based on a series of methylation profiles in the GEO dataset. As shown in Figure 1B, this workflow involved five stages. (1) The AD, PD, PSP, FTD, and normal aging markers were identified by machine learning methods, respectively. (2) The aging score and disease score were given accordingly, thus the accelerated aging pattern in ND was validated. (3) According to the correlation between each pair of genes and the aging score, the aging acceleration differential network was thereby constructed. (4) Both network and enrichment analysis were be used to explore the mechanisms that relate accelerated aging to ND, respectively. (5) Sensitivity analysis was then performed to further investigate cellular homeostasis imbalances in different ND types using the Markov Chain Monte Carlo (MCMC) method.

## Results

### Modeling the Aging and Disease Predictor and Identifying Relative Risk Markers

**FIGURE 2:**
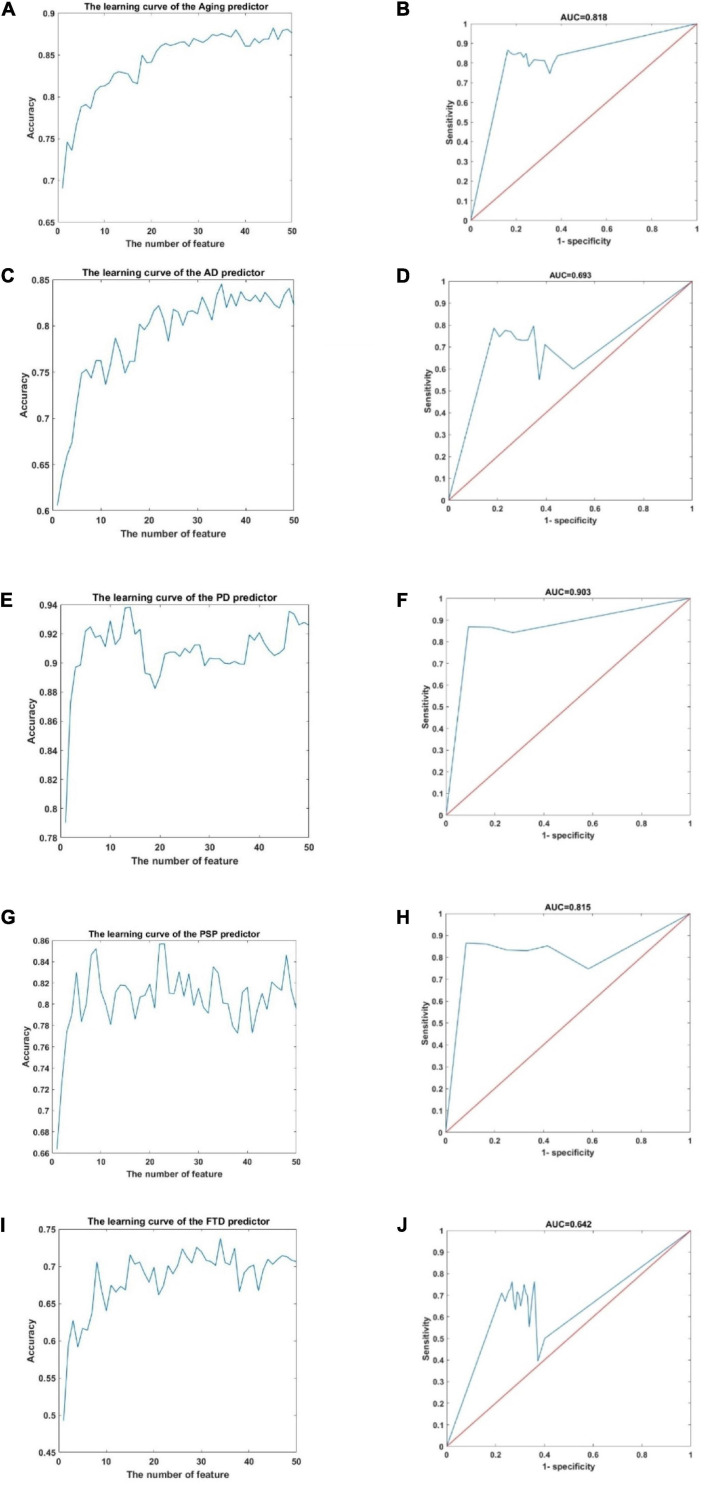
The learning curve and ROC curve in the aging predictor and improved PD predictor. **(A)** The learning curve of the aging predictor. **(B)** The ROC curve of the aging predictor. **(C)** The learning curve of the AD predictor. **(D)** The ROC curve of the AD predictor. **(E)** The learning curve of the PD predictor. **(F)** The ROC curve of the PD predictor. **(G)** The learning curve of the PSP predictor. **(H)** The ROC curve of the PSP predictor. **(I)** The learning curve of the FTD predictor. **(J)** The ROC curve of the FTD predictor.

DNA methylation profiles were used in this work, more specifically, 22905 cpg sites from 6 GSE profiles, the details of which are shown in Supplementary Text 1, 2. The aging predictor and disease predictor were modeled by the classification ensemble algorithm of the classification tree (shown in detail in the section “Materials and Methods” below), and the best predictor was selected based on 10-fold cross-validation (Table 1 and Supplementary Text 1). The learning curves of the training data set are shown in Figure 2.

**TABLE 1:** Accuracy of aging and disease predictors.

	**The aging predictor**	**The AD predictor**	**The PD predictor**	**The PSP predictor**	**The FTD predictor**
Training dataset accuracy	0.8823	0.8453	0.9383	0.8675	0.7374
Test dataset accuracy	0.8272	0.7302	0.9331	0.8568	0.7092

According to the cross-validation results, the top 46, 35, 14, 23, and 34 dimensions of cpg sites were identified as risk biomarkers related to aging, AD, PD, PSP, and FTD, respectively. Next, these five prediction results were verified in the independent test data set. This ensured that the classification results in the test data had adequate accuracy (Table 1). The receiver operating characteristic (ROC) curves of the predictors are also shown in Figure 2. The area under the ROC curve (AUC) 0.818, 0.693, 0.642, 0.903, and 0.857 in the predictor of aging for AD, PD, PSP, and FTD, respectively. Overall these results showed that the predictor had high accuracy and efficiency.

It has been reported that the identified biomarkers are closely related to aging and ND (Table 2; Salmerón et al., 2001; Hu et al., 2004; Katoh, 2008; Qiu and Ghosh, 2008; Fujimoto et al., 2009; Su et al., 2009; Kozarova et al., 2011; Euskirchen et al., 2012; Berwick and Harvey, 2014; Lopez-Pelaez et al., 2014; Alquézar et al., 2016; Chailangkarn et al., 2016; Densham et al., 2016; Husain et al., 2016; Pîrşcoveanu et al., 2017; Kurihara et al., 2019; Monteiro and Forti, 2019). Interestingly, these related functions (i.e., DNA damage repair response, mitochondrial dysfunction, or Ca ion homeostasis disorder), further indicated the imbalance of cellular homeostasis, and abnormal cell death was further induced across various ND types. Therefore, cellular homeostasis disorder may not only disrupt normal brain functioning but also lead to pathological changes related to ND.

**TABLE 2:** The top risk markers of aging and ND.

	**Gene Index**	**Gene Symbol**	**Function**	**References**
Aging	cg20692569	FZD9	(1) Through the typical signaling pathway of β – Catenin, the cell cycle arrest is negatively regulated to inhibit neuronal apoptosis, which plays a role in the survival of neural progenitor cells	Katoh, 2008; Fujimoto et al., 2009; Berwick and Harvey, 2014; Chailangkarn et al., 2016
			(2) Cell proliferation and cell movement are significantly inhibited	
			(3) Regulating the WNT signaling pathway.	
AD	cg12411068	SMARCA4	(1) Chromatin remodeling is involved in the transcriptional activation and inhibition of selection genes	Qiu and Ghosh, 2008; Su et al., 2009; Euskirchen et al., 2012; Husain et al., 2016
			(2) SWI/SNF is a component of the chromatin remodeling complex, which performs key enzyme activities and alters the chromatin structure by changing DNA histone contact in the nucleosome in an ATP dependent manner (i.e., chromatin remodeling enzymes play an important role in gene expression, DNA replication and repair, cell division and other biological processes)	
			(3) As the component of CREST – BRG1	
			(4) Modulating calcium dependence of complexes	
			(5) SMARCA4 promoted the self-renewal/proliferation of neural stem cells	
PD	cg16414945	DUSP12 (HYVH1)	(1) Playing a role in cell survival and ribosome biosynthesis	Kozarova et al., 2011; Pîrşcoveanu et al., 2017; Monteiro and Forti, 2019
			(2) Regulating the cell cycle	
			(3) As a key factor in dephosphorylation of tyrosine and serine/threonine residues	
			(4) Phosphorylating the Tau protein with multiple serine/threonine and tyrosine phosphorylation sites, which was an important indicator of PD	
PSP	cg01994328	BRCA1	(1) Regulating the DNA double strand break repair pathway, or leading to the DNA damage	Densham et al., 2016; Kurihara et al., 2019
			(2) Mis-localization of BRCA1 was associated with tau aggregation and the DNA damage	
			(3) Involved in the pathogenesis of PSP	
FTD	cg04223844	IKBKB (IKKB/IKK)	(1) Phosphorylation of IKK related kinases can prevent excessive production of inflammatory mediators and TNF mediated RIPK1 dependent cell death	Salmerón et al., 2001; Hu et al., 2004; Lopez-Pelaez et al., 2014; Alquézar et al., 2016
			(2) IKBKB can affect the NF-κB signaling pathway, which was the reason for FTD	

### Comparison of Aging Scores Between ND and Normal Aged Samples

**FIGURE 3:**
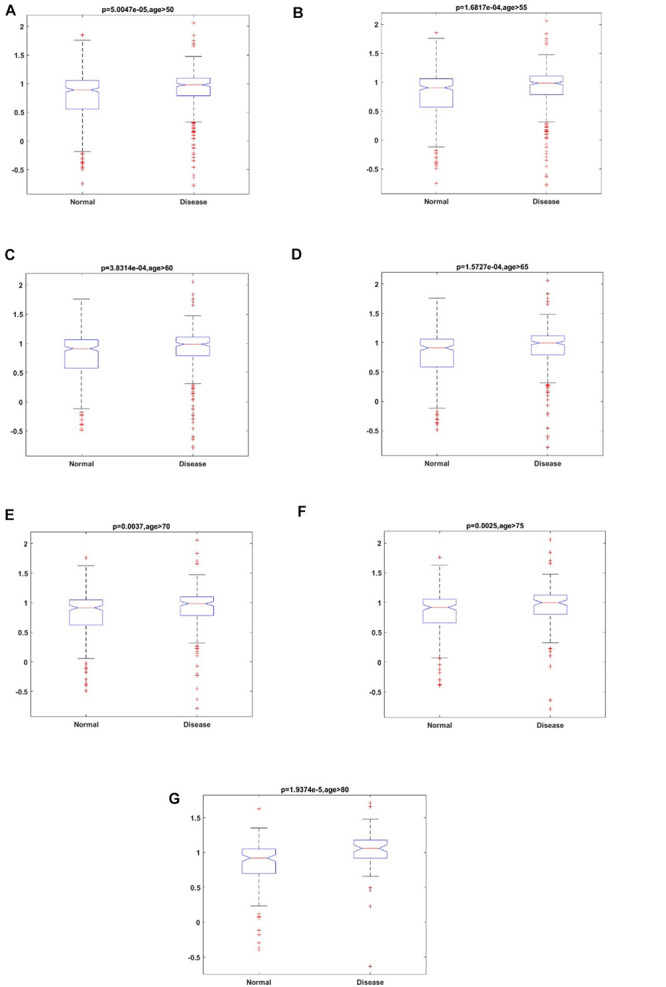
The results of the Kruskal–Wallis test for aging scores of ND individuals and normal individuals in different age groups. **(A)** Age ≥ 50; **(B)** Age ≥ 55; **(C)** Age ≥ 60; **(D)** Age ≥ 65; **(E)** Age ≥ 70; **(F)** Age ≥ 75; and **(G)** Age ≥ 80.

To study the accelerated aging pattern in ND, the aging score was calculated based on 46 aging risk biomarkers (shown in the section “Materials and Methods” below). Both the median and median scores for different age groups are shown in Table 3. With the increase in age, the aging scores in both disease and normal aged individuals showed an upward trend. The results also showed that the accelerated aging pattern in ND compared with the normal aged people, which was consistent with previous studies (Liu et al., 2020). Compared with the chronological age, the aging score was more informative. The Kolmogorov–Smirnov (K–S) test was used to test the aging scores of each ND type as well as the healthy aged sample coming from the normal distribution. The results showed that the *p*-value was close to 0 and rejects the original hypothesis (shown in Supplementary Table 1). Consequently, the Kruskal–Wallis test was then executed to verify whether the aging score reflected a significant difference between ND and normal individuals, where different age groups, as well as different ND types, were compared. The results are shown in Figure 3, Table 3, Supplementary Figure 1, and Supplementary Table 2. These results indicate that the aging scores of ND sample individuals exhibit a significantly accelerated aging pattern, compared with those of normal aged individuals (*p* < 0.05).

**TABLE 3:** The chronological age and aging scores of ND and control in different age groups.

**Age**	**The median age in ND**	**The median age in control**	**The mean age in ND**	**The mean age in control**	**The median aging score in ND**	**The median aging score in control**	**The mean aging score in ND**	**The mean aging score in control**
≥50	71	72.19	71	72.21	0.978332	0.890503	0.889628	0.782356
≥55	75	74.42	72	73.06	0.9834	0.903807	0.891899	0.792415
≥60	77	77.59	73	74.12	0.986455	0.911084	0.896285	0.802538
≥65	77	79.02	75	76.26	0.993786	0.911595	0.91115	0.802051
≥70	82	82.47	78	79.53	0.985051	0.914107	0.906385	0.814409
≥75	85	85.07	81	82.48	1.000348	0.919098	0.945376	0.826603
≥80	87	87.70	85	86.56	1.058764	0.919653	1.026904	0.82923

### The Aging Acceleration Differential Network Provided Insights Into the Key Biological Functions of ND

**FIGURE 4:**
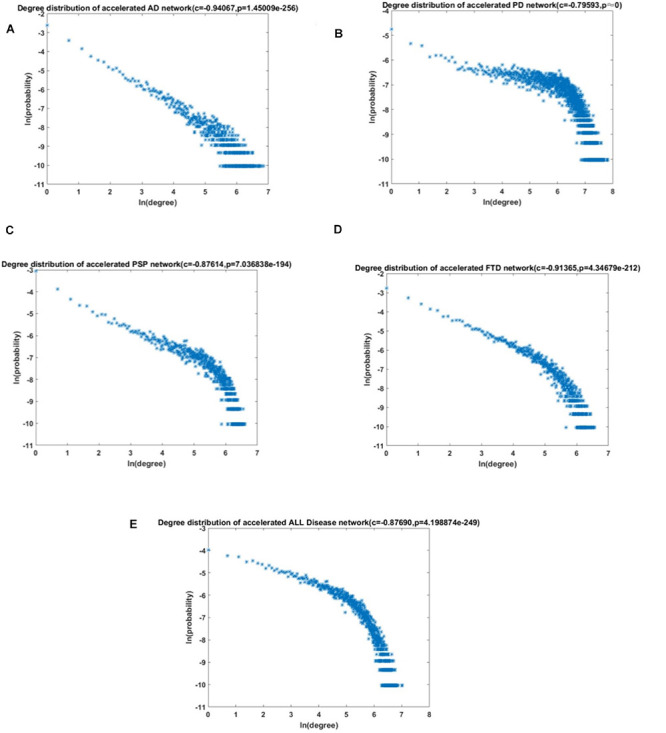
The degree distribution of the acceleration aging network. **(A)** AD; **(B)** PD; **(C)** PSP; **(D)** FTD; and **(E)** All NDs.

To better study the potential mechanisms between aging and ND, the aging acceleration differential network was constructed, where FDR < 0.1 was used to ensure reliable correlation. The (partial) coefficients were also summarized and used to compare the relationship of each pair of cpg cites in the context of aging. Aimed at verifying the scale-free characteristics, the probability corresponding to logarithmic transformation and its degree were used to test the power-law distribution (shown in the section “Materials and Methods”). The node degree distribution curve and Pearson correlation coefficient of different ND types are shown in Figure 4.

The results revealed that the aging acceleration network was with the scale-free pattern. The degree and frequency were inversely correlated, and only a small proportion of genes had a high degree. Furthermore, the Fisher’s exact test was carried out to calculate the similarity between the different networks based on the training data and the test data, respectively, and the result showed a *p*-value very close to 0 (1e-23238, 1e-131120, 1e-3103, 1e-44540, and 1e-139070 for AD, PD, PSP, FTD, and all NDs, respectively). In addition, the node with the highest degree in the network was also significantly correlated with maintaining cellular homeostasis (Table 4; Crowe et al., 1994; Dong et al., 1997; Keats et al., 2007; Tang et al., 2011; Kaneko et al., 2016; Kula et al., 2019; Anerillas et al., 2020).

**TABLE 4:** Network markers of the highest degree.

	**Gene Index**	**Gene Symbol**	**Degree**	**Function**	**References**
AD	cg09414535	GRIP1	945	GRIP1 mediates synaptic and non-synaptic signals. It plays an important role in the development and function of oligodendrocytes and their precursors *in vivo*	Kula et al., 2019
PD	cg11672225	RNF185	2478	(1) E3 ubiquitin ligase regulates selective mitochondrial autophagy by mediating “lys-63” linked multi ubiquitination of BNIP1	Tang et al., 2011; Kaneko et al., 2016
				(2) Plays a role in the endoplasmic reticulum related degradation pathway, which targets misfolded proteins accumulated in the endoplasmic reticulum for ubiquitination and subsequent proteasome mediated degradation, and protects cells from endoplasmic reticulum stress-induced apoptosis	
PSP	cg14448116	GPR21	720	A constitutively active receptor that can be coupled with Gαq type G protein, leading to the activation of mitogen activated protein kinase, thus inducing cell senescence, growth arrest, and cell death	Anerillas et al., 2020
FTD	cg15784615	LTBR	706	(1) Promoting apoptosis through TRAF3 and TRAF5	Crowe et al., 1994; Keats et al., 2007
				(2) Leading to constitutive activation of the non-canonical NF-κB pathway, which is involved in cellular immune response, growth control, and apoptosis	
ND	cg09414535	GRIP1	1114	Acting as local scaffolds for the assembly of multi protein signaling complexes and mediators for the transport of their binding partners at specific subcellular locations of neurons	Dong et al., 1997

### Underlying ND Mechanisms Based on the Enrichment Analysis in the Aging Acceleration Differential Network

**FIGURE 5:**
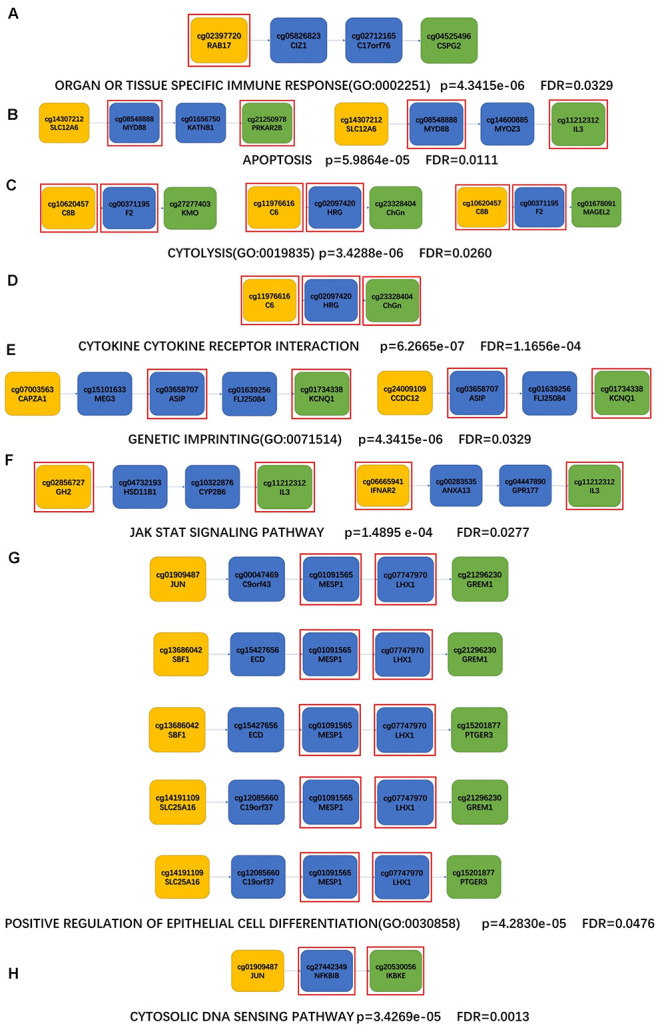
The shortest paths for enrichment analysis of KEGG and BP. **(A,B)** AD enrichment results; **(C,D)** PD enrichment results; **(E,F)** PSP enrichment results; **(G,H)** FTD enrichment results; **(A,C,E,G)** enriched KEGG pathway; **(B,D,F,H)** enriched BP terms; The yellow nodes represent the aging biomarkers, the blue nodes represent the genes connecting aging biomarkers and ND biomarkers, the green nodes represent the ND biomarkers, and the genes in the red square frames coincide with those genes in the enriched functions.

To further investigate the potential mechanisms between aging and ND, each shortest path of aging-AD, aging-PD, aging-PSP, and aging-FTD were identified based on the aging acceleration differential network using the Dijkstra algorithm. Enrichment analysis was then performed based on each of the shortest paths, the special functions (i.e., the Kyoto Encyclopedia of Genes and Genomes (KEGG) pathway, and the Biological Process (BP) term in Gene Ontology (GO), shown in Figure 5 and Table 5). These indicated multiple dysfunctions of different ND types (McGeer et al., 1989; Behl, 2000; Nagatsu et al., 2000; Everse et al., 2011; Obulesu and Lakshmi, 2014; Caputi and Giron, 2018; Cui and Xu, 2018; Yan et al., 2018; Liu et al., 2019; Porro et al., 2019; Starr, 2019; Ashrafizadeh et al., 2020; Burgaletto et al., 2020; Luo et al., 2020; Paul et al., 2021), respectively.

**TABLE 5:** Enrichment analysis results (minimum *p*-value and FDR) of the four disease enrichment pathways are observed, respectively.

**ND**		**Name**	***P*-value**	**FDR**	**Function**	**References**
AD	KEGG	APOPTOSIS	5.9864e-05	0.0111	Excessive apoptosis can lead to AD	Behl, 2000; Obulesu and Lakshmi, 2014
	BP	ORGAN OR TISSUE SPECIFIC IMMUNE RESPONSE (GO:0002251)	4.3415e-06	0.0329	(1) Complement proteins are involved in the pathogenesis of Alzheimer’s disease by attaching to diseased tissues or activating cells related to the immune system	McGeer et al., 1989; Burgaletto et al., 2020
					(2) Neuroinflammatory response promotes the progression of neurodegeneration in AD	
PD	KEGG	CYTOKINE-CYTOKINE RECEPTOR INTERACTION	6.2665e-07	1.1656e-04	In PD patients, dopamine is induced by programmed cell death (apoptosis) caused by increased levels of cytokines.	Nagatsu et al., 2000
	BP	CYTOLYSIS (GO:0019835)	3.4288e-06	0.0260	Cytochrome C in the form of peroxidase catalyzes cell lysis, which leads to the death of neurons in PD	Everse et al., 2011
PSP	KEGG	JAK STAT SIGNALING PATHWAY	1.4895 e-04	0.0277	(1) Involved in many biological processes such as cell proliferation and apoptosis	Yan et al., 2018; Liu et al., 2019; Porro et al., 2019; Ashrafizadeh et al., 2020
					(2) Overactivation mediates the imbalance of intracellular homeostasis and leads to premature aging	
					(3) JAK/STAT plays an important role in the development and function of innate and adaptive immunity	
					(4) Abnormal activation of the JAK/STAT pathway is obvious in neuroinflammatory diseases	
					(5) When the pathway is abnormally activated, it regulates the anti-inflammatory response of microglia	
	BP	GENETIC IMPRINTING (GO:0071514)	4.3415e-06	0.0329	(1) DNA methylation is significantly correlated with neurodevelopment and neurodegeneration	Cui and Xu, 2018; Starr, 2019
					(2) Plays an indispensable role in adult learning, memory, and cognition	
					(3) Can affect age-related cognitive function and the occurrence and progress of ND.	
FTD	KEGG	CYTOSOLIC DNA SENSING PATHWAY	3.4269e-05	0.0013	(1) There is cytoplasmic DNA in macrophages, which has a significant effect on the activation of macrophages	Luo et al., 2020; Paul et al., 2021
					(2) When cytoplasmic DNA is detected, the signal is transmitted through CGAs penetration pathway	
					(3) CGAs usually mediate immune monitoring and neuroprotection, but excessive involvement will cause damage to the nervous system	
	BP	POSITIVE REGULATION OF EPITHELIAL CELL DIFFERENTIATION (GO:0030858)	4.2830e-05	0.0476	Intestinal epithelial cells are used to balance the innate immune response, thus affecting the early stage of the subsequent neurodegenerative cascade	Caputi and Giron, 2018

**FIGURE 6:**
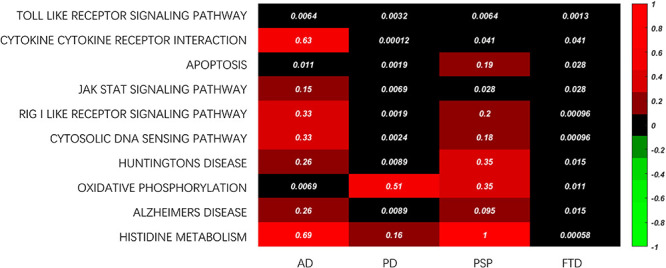
The common enriched KEGG pathway across ND types.

In addition, common enriched functions were investigated to study the crucial mechanisms across different ND types, where the top ten KEGG pathways are shown in Figure 6. For example, the Toll-like receiver (TLR) signaling pathway was the top KEGG pathway related to all four ND types. The TLRs activated their downstream pathways and then induced NF-κB and Pro-Il-1β, both of which are related to neuroinflammation and the pathogenesis of a variety of neurological diseases (Azam et al., 2019). Furthermore, the activation of TLRs can also induce the inflammatory response of macrophages by activating the transcription cascade (Lauterbach et al., 2019). The inflammatory response induced by activation of TLRs is considered to be closely related to AD, PD, PSP, and FTD (López González et al., 2016). Therefore, the importance of the Toll-like receiver signaling pathway is reflected in a variety of ND types. Furthermore, cytokine receptor interaction is also critical in ND progression. For example, microglia can release cytokines to trigger a cellular inflammatory response and participate in the occurrence and development of NDs (Colonna and Butovsky, 2017). Cellular apoptosis is also a fundamental process in the progression of nervous system diseases (Radi et al., 2014).

### The Sensitivity Analysis Further Revealed Crucial Cellular Homeostasis Imbalances Across ND Types

In order to further investigate the complex cellular homeostasis imbalances across different ND types, a global sensitivity analysis was performed using the MCMC method (shown in section “Materials and Methods”). To study the relationship between aging and ND markers, the differential MCMC K–S statistics were calculated based on each “aging-ND” pair; then the top ranked “aging-ND” pairs were identified for each disease (as described in the section “Materials and Methods”). The aging markers shared by different ND types as well as the disease markers within each specific ND type were then explored, based on both aging and ND MCMC differences.

TSC2 was the risk marker with the highest absolute MCMC difference across the four types of ND. It has been reported that the phosphorylation of TSC2 could restore the expression of NF-κB protein, which is vital to reduce the production of inflammatory mediators and ND markers (Kumar et al., 2017). In addition, the aging markers identified in all four diseases were identified according to the obtained “aging-ND” pairs. Among them, there were two aging markers with high frequencies. CHGN-1 (CSGALNACT1, frequency = 40) is a key enzyme for the production of CSPGs, participating in the demyelination, remyelination, axonal degeneration, and regeneration of the central nervous system (Saigoh et al., 2016). SLC15A2 (PEPT2, frequency = 24) can affect the production of pro-inflammatory cytokines by macrophages (Gupta et al., 2013). The excessive release of pro-inflammatory cytokines causes neuronal damage (Shen et al., 2004).

In AD, CDCA7L was with the highest absolute MCMC difference. It was associated with the intelligence quotient (Pan et al., 2011), which could even inhibit the Monoamine oxidase A(MAOA) promoter as well as neuronal cell death (Ou et al., 2006).

EFNB2 had the highest absolute MCMC difference in PD. EFNB2 was involved in adjusting the development of the nervous system and the neuronal migration (Lévy et al., 2018), and could also activate the Eph/efn forward signaling pathway as well as cell apoptosis (Zhong et al., 2019).

In PSP, the marker with the highest absolute MCMC difference was BCL2, which could affect the activity of the mitochondrial complex (Chong et al., 2020). BCL2 could also enhance the anti-apoptotic effect of nerve growth factor (NGF) and promote the survival and differentiation of nerve cells (Troullinaki et al., 2019).

In FTD, MICAL1 had the highest absolute MCMC difference. Through interacting with STK38 and STK38L, it acted as a negative regulator of cell apoptosis (Zhou et al., 2011). MICAL1 is also involved in the regulation of lamina specific connections in the nervous system (Schmidt et al., 2008).

### The Network Marker Revealed Critical Mechanisms Between Aging and ND

**FIGURE 7:**
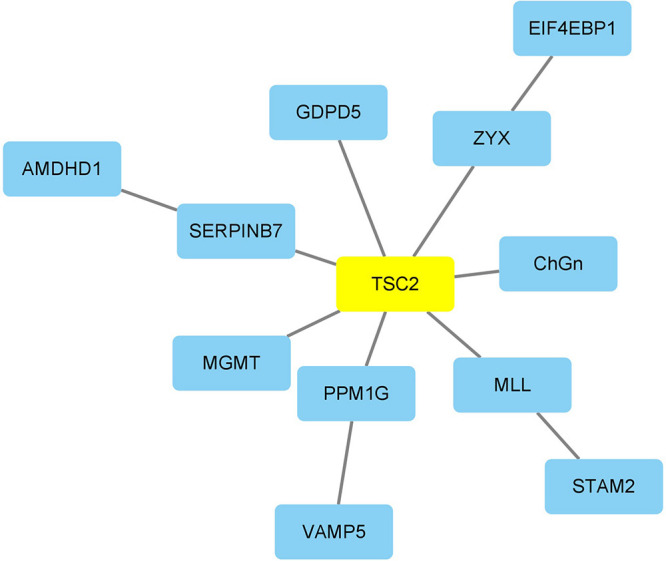
The network marker with the highest betweenness of ND.

The potential network markers were also found out based on each “aging-ND” pair from the results of sensitivity analysis by summarizing the betweenness in the aging acceleration differential network, as shown in Table 6, Figure 7, and Supplementary Figure 2. As a result, DUSP12 had the largest betweenness across the four ND types. DUSP12 could regulate the c-Jun N-terminal kinase (JNK) signaling pathway by dephosphorylating its substrate, which was critical to cell differentiation, apoptosis, and other neural functions in ND progression (Ha et al., 2019). It also has been reported that the overexpression of DUSP12 inhibited the production of pro-inflammatory cytokines and chemokines (Cho et al., 2017).

**TABLE 6:** The top network markers.

	**cpg index**	**Gene symbol**	**Betweenness**	**Permutation *p*-value**
D	cg02198582	TSC2	6	0
	cg07044282	ANGPTL1	6	0
	cg14643978	TMC1	5	0
	cg26660631	FLJ32011	5	0
	cg00513467	KLHL9	4	0.002
	cg00594952	RIMS3	4	0
	cg01430807	NDUFB8	3	0
	cg04183425	ASF1A	3	0
	cg04682845	TMEM42	3	0
	cg05347567	ZC3H10	3	0
AD	cg10559803	RALGPS2	37	0
	cg08959992	PCBP2	37	0
	cg00032227	NAT9	37	0
	cg12085660	C19orf37	37	0
	cg13629753	GBP2	37	0
	cg14191109	SLC25A16	37	0
	cg21049762	TCIRG1	37	0
	cg24705286	JMJD5	37	0
	cg05040447	QP-C	36	0
	cg01869233	C20orf75	31	0
PD	cg10303487	DPYS	76	0
	cg00003994	MEOX2	69	0
	cg00107187	FLJ42486	69	0
	cg01078434	MAS1L	65	0
	cg00497084	PPEF1	52	0
	cg03245641	GPHA2	43	0
	cg05019661	ADK	40	0
	cg02748539	SLC9A3	39	0
	cg03993463	KCNJ15	39	0
	cg07356189	CXorf2	39	0
PSP	cg04145477	QRSL1	37	0
	cg00614413	PTPRS	35	0
	cg00834796	JAKMIP2	35	0
	cg12347740	MGC34647	35	0
	cg20252016	CDCA5	35	0
	cg00093177	FLJ43826	34	0
	cg05628549	PRKCDBP	33	0
	cg17720231	IGSF9	33	0
	cg18139769	SGCE	33	0
	cg11655691	CBARA1	32	0
FTD	cg04089739	C3	51	0
	cg23756219	DRP2	50	0
	cg15427656	ECD	45	0
	cg24921089	AMPD3	45	0
	cg19177941	ALDH1A3	43	0
	cg02982734	MAGEL2	42	0
	cg17241657	C4orf16	42	0
	cg02800334	ANXA13	41	0
	cg12885244	LOC51315	41	0
	cg12796229	C18orf43	36	0

Furthermore, the genes (cpg site) within each shortest “aging-ND” path were also identified. For example, the top network (betweenness) marker in AD was RALGPS2. It has hitherto been revealed that RALGPS2 silencing induces cell apoptosis, by improving cell cycle inhibitors p27 and p21 (Santos et al., 2016). The top network marker in PD was MEOX2 (GAX). The deletion of MEOX2 can lead to decreased capillary density as well as resting cerebral blood flow, and then promote the loss of hypoxic angiogenesis in the brain. It causes hypoxia reaction and cell death (Wu et al., 2005). QRSL1, whose mutation may lead to dysfunction of mitochondrial energy production and mitochondrial disorder (Albers and Beal, 2002), was the top network marker in PSP. The top network marker in FTD was C3. C3 uptake by cells reduces stress-related cell death (e.g., oxidative stress or starvation). In the process of inflammation, storing C3 in cells can prevent some substances that induce cell death (Kulkarni et al., 2019). In brief, these network markers were also informative and indicate the relationship between cellular homeostasis and NDs.

## Discussion

In this article, a series of computational methods were integrated to explore the potential mechanisms between aging and four types of ND, as well as the commonalities and specificities of these four types of ND based on aging acceleration. Firstly, aging markers and ND markers were identified by the aging predictor and disease predictor, respectively. Secondly, according to the selected aging markers, the aging score of dementia patients showed an accelerated aging pattern compared with normal aged people. Furthermore, the aging acceleration differential network was constructed based on the aging score, then crucial shortest “aging-ND” paths were discovered.

The aging markers (using MCMC) indicated that an imbalance in cellular homeostasis is the key bridge, linking accelerated aging and ND. For example, the top aging marker was TSC2, as a risk marker of ND by affecting the inflammatory response (Kumar et al., 2017). Furthermore, other aging markers revealed the critical roles of cellular homeostasis in ND progression. CHGN (CSGALNACT1) and SLC15A2. CHGN-1 are the critical enzymes for CSPG production (Saigoh et al., 2016), which is the main pericellular and extracellular component of the regulatory environment (Hu et al., 2018). SLC15A2 (PEPT2) plays a key role in regulating the concentration of neuropeptides in extracellular fluid (Dheen et al., 2007).

The top risk marker in the AD predictor was SMARCA4, which participated in cellular biological processes by altering the contact of DNA histone in the nucleosome (Euskirchen et al., 2012). RALGPS2 was with the largest betweenness in AD and as a potential pathogenic index of AD (Liu et al., 2007). The marker with the highest absolute MCMC difference in AD was CDCA7L, as the regulator of caspase-3, which played a significant role in cell death progression (Yosefzon et al., 2018). The enrichment result of AD indicated apoptosis. Our results emphasize that cellular homeostasis disorder in patients with AD may be due to the imbalance of intracellular calcium homeostasis and abnormal DNA repair, resulting in excessive cellular apoptosis and then leading to AD.

DUSP12 was the top risk marker in the PD predictor, as a regulator of the cell cycle (Kozarova et al., 2011). MEOX2 was the top network marker in PD, leading to the loss of neurons and a significant reduction in the microvessels associated with plaque, which further indicated the synergistic effect of vascular compromise and amyloid deposition on the dysfunction of neurons (Soto et al., 2016). EFNB2 (with the maximum differential MCMC value) is closely related to the cellular autophagy pathway (Zhong et al., 2019); it could also activate the quiescent static stem cells and promote the depletion of cells (Ottone et al., 2014). Enrichment results identified the cytokine receptor interaction involved in many cellular processes, including cell growth, cell differentiation, cell apoptosis, cellular homeostasis, and so on. Our results suggest that the disorder of cytokines, oxidative stress, and protein deposition may lead to disorder of homeostasis and cell death, and then trigger the occurrence of PD.

The results indicate that BRCA1 was the top risk marker in PSP. The high expression of BRCA1 can affect the DNA self repair pathway and cause DNA damage (Densham et al., 2016). In addition, the top network marker (with the highest betweenness) was QRSL1. The mitochondrial dysfunctions induced by QRSL1 play an important role in advanced PSP (Albers and Beal, 2002). BCL2 was with the highest absolute MCMC difference in PSP, which could prevent oxidative stress-induced DNA damage as well as cell death (Chong et al., 2020). It is also the key regulator of cell apoptosis (Roberts, 2020). In summary, DNA damage, advanced neuroinflammation, and cell death induce the occurrence of PSP.

The top FTD risk marker IKBKB plays an important role in the NF-κB signaling pathway (Salmerón et al., 2001), which is the main cause of FTD (Alquézar et al., 2016). Furthermore, MICAL was with the highest absolute MCMC difference in FTD, playing an important role in cellular redox regulation, survival, development, and death (Ortegón Salas et al., 2020). C3 was the top network marker in FTD. The content of C3 in FTD was significantly increased (Katzeff et al., 2020). The enrichment results showed that the cytosolic DNA sensing pathway can damage the nervous system. Therefore, based on the above studies, FTD is considered the imbalance of intracellular inflammatory factors and excessive oxidative stress, leading to an imbalance of cellular homeostasis.

The extracellular fluid, cellular metabolisms, and inflammatory response were considered the common characteristics of the cellular homeostasis imbalance across different types of ND in the context of aging acceleration. Another study found that the extracellular fluid of microglia can affect cellular homeostasis and cell survival (Erny et al., 2015). Cell metabolites are also crucial for cellular homeostasis (Koo and Guan, 2018). Cellular inflammation can trigger a cellular stress response. When neuroinflammation occurs, extracellular fluid is considered the key index of cellular homeostasis imbalance. There is various evidence to suggest that many types of ND are related to the inflammatory response (Stephenson et al., 2018). Moreover, these risk factors interact with each other and promote the development of NDs.

**FIGURE 8:**
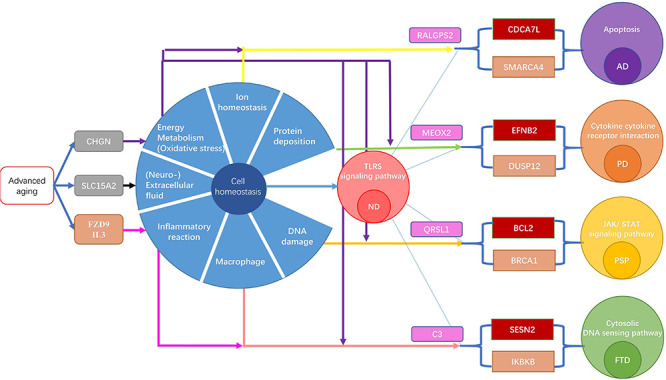
The mechanism of ND induced by acceleration aging. The brown gene is a biomarker of ND. The gray gene is the top MCMC marker of aging. The red gene is the top MCMC marker of each ND type. The pink gene is the network node with highest betweenness. The yellow arrow indicates the connection of Calcium homeostasis. The purple arrow represents a link to DNA damage repair response. The green arrow represents the link with the mitochondrial function. The brown arrow represents a link to oxidative stress. The black arrow indicates the connection with ubiquitin. The red arrow indicates the connection of the intracellular signal.

In cell death and neurodegenerative theory, the dynamic balance of neurons is induced by advanced brain aging, and accompanied by pathological changes (Andreone et al., 2020), such as mitochondrial dysfunction, and oxidative stress, which are causes that drive advanced cell apoptosis. The abnormal apoptosis of nerve cells then leads to a decline in normal function and eventually the occurrence of ND. Our results also found critical mechanisms of cellular homeostasis imbalance in different ND progressions, thus both the common and specific cellular homeostasis imbalances are summarized. Moreover, these results indicate the critical mechanisms of cellular homeostasis imbalance in different ND types, thus both the common and specific cellular homeostasis imbalances were summarized (Figure 8). Interestingly, the common characteristics were identified as extracellular fluid, cell metabolism, and the inflammatory response, based on aging acceleration. In terms of specific characteristics, abnormal calcium homeostasis and the DNA repair pathway damage lead to advanced cell death and then promote the occurrence of AD. This may be due to abnormal protein deposition, oxide accumulation, and cytokine disorder (related to advanced oxidative stress), leading to the progression of PD. DNA damage and mitochondrial dysfunction induce PSP. Abnormal cellular differentiation or the overloading of cytoplasmic DNA in macrophages triggers extra inflammatory factors in FTD.

## Conclusion

The present study used machine learning methods to identify the risk markers of different types of ND and the normal aging process for each disease, respectively. The aging score was thereby summarized. The results showed that ND sample individuals exhibited significantly accelerated aging patterns. By comparing the correlation of each pair of cpg sites between ND and the normal aged group, aging acceleration differential networks were constructed. In addition, the mechanisms of cellular homeostasis imbalances across different ND types were found based on enrichment analysis and sensitivity analysis. The results showed that in the background of accelerated aging, extracellular fluid, cell metabolism, and the inflammatory response induce imbalances in cellular homeostasis, which trigger ND progression. The specific mechanisms of AD, PD, PSP, and FTD were also identified, including Ca ion disorder, protein deposition, DNA damage, and dysfunctions in macrophages, respectively.

## Materials and Methods

### DNA Methylation Profiles and Data Pre-processing

The DNA methylation profiles were obtained from the Gene Expression Omnibus (GEO) database (Supplementary Table 1), including GSE15745, GSE51923, GSE53740, GSE57361, GSE66351, and GSE138597, along with the age index. These datasets were from seven different platforms: GPL6104, GPL8178, GPL8490, GPL5175, GPL13534, GPL 11154, and GPL 21145.

The steps of obtaining DNA methylation profiles were as follows:

(1) cpg sites with missing values ≥ 30% were deleted.
(2) According to different brain regions, the k-nearest neighbor algorithm (*k* = 10, with the Euclidean distance) was used to supplement the missing values.
(3) Individuals without the age index were deleted.
(4) Patients with early onset ND (age ≤ 50) were removed.
(5) *z*-score normalization was performed based on the healthy aged individuals.
(6) The Singular Value Decomposition (SVD) method was used to eliminate inter-sample variation based on the top three principal components in healthy aged individuals.
(7) The *z*-score was then utilized to normalize all individuals based on the mean and standard deviation of healthy aged individuals.

The final sample included 366 healthy youth samples (age ≤ 50, 250, in the training data and 116 in the test data), 442 normal old individuals (age > 50, 300+142), 128 AD individuals (85+43), 36 PD individuals (25+11), 123 FTD individuals (85+38), and 42 PSP individuals (30+12). The DNA methylation data set included 22905 cpg sites (Supplementary Text 2).

### Modeling the Aging Predictor as Well as Each ND Predictor

After randomization as well as a random disorder, the healthy population samples were divided into training data set and test data set. The ratio of training data set samples to test data set samples were close to 2:1. The ReliefF algorithm was used to select key features, then the first 50 models were studied to train predictors. The optimal model was selected by 10-cross validation. To verify the accuracy of the aging predictor, the selected model was verified in the test data set.

(1) The normal aged group (age > 50) were labeled as 1 and the young healthy group (age ≤ 50) were labeled as 0.

(2) The 22,905 cpg sites were sorted by the ReliefF algorithm.

(3) The predictor was generated using the ensemble learning algorithm.

The ensemble learning algorithm established 100 decision tree models, and then put the data in the 100 classifiers for decision-making. The classification result was optimized to get the most answers from the 100 classifiers. For 100 classifiers, weak weight was given to the classifiers with more wrong classification results using the formula of the weight coefficient. The calculation method was as follows:

(1) $$D_{n}=D_{n-1}\times \frac{1-\varepsilon_{n}}{\varepsilon_{n}}\left(D_{1}=\frac{1}{m}\right)$$

(2) $$\varepsilon_{n}=\sum D_{n}||y_{i\neq h_{i}\left(x_{i}\right)}$$

and the classifier weight was:

(3) $$\alpha_{n}=\frac{1}{2}ln\left(\frac{1-\varepsilon_{n}}{\varepsilon_{n}}\right)$$

where *m* was the number of predicted variables; *D*_*n*_ was the weight of each sample;

ε*_*n*_* was the classifier error; *x*_*n*_ was the member of predicted variables; *y*_*n*_ represented the corresponding attribute value of *x*_*n*_ and *h*_*n*_ was each attribute.

One hundred weak classifiers were superimposed to generate the strongest classifier. The optimal model was selected by 10-fold cross validation. Ultimately, the model with the highest accuracy rate was chosen. The identified features were considered as aging and each ND marker, respectively.

To construct disease predictors, 442 healthy aged individuals, 128 AD individuals, 36 PD individuals, 123 FTD individuals, and 42 PSP individuals were randomly chosen. In the classification process of each disease predictor, the ND sample was labeled as 1 and the normal aged sample was labeled as 0. To avoid the unbalance of samples in machine learning, the normal aged group (training data) were divided into 3, 9, 7, and 3 subgroups to compare with AD, PD, PSP, and FTD (Supplementary Table 2), respectively.

### Calculating the Aging Score

For each sample, the aging score was calculated as follows:

(1) The regression result (from young as 0 to old as 1) was used as the aging score using the ensemble learning algorithm from 100 regression tree models based on aging markers.
(2) The K–S test was used to test whether the aging score came from the normal distribution. The *p*-value was the least significant level to reject the original hypothesis. The smaller the *p*-value, the easier it was to reject the original hypothesis. Both the original aging score and the score adjusted by the transformation of the chronological age were tested, where the chronological age was transformed using the sigmoid function:

(4) $$transformatedage=\frac{1}{1+exp\left(-\left(age-50\right)/50\right)}$$

The transformed age was predicted based on the aging scores using linear regression:

(5) $$Adjusted\_score=b^{*}aging\_score-transformatedage$$

where *b* is the regression coefficient for aging score.

(3) The Kruskal–Wallis test was used to compare the accelerated aging pattern between ND and normal aged individuals for different age groups.
(4) In addition, the risk score of each ND predictor was also calculated for further network analysis.

### Constructing the Aging Acceleration Differential Network

To further reveal the relationship between aging and ND, the aging acceleration network was constructed based on the training data set and test data set.

(1) To compare the relationship of each pair of cpg sites in the context of the aging process, both the Pearson correlation coefficient for each pair of cpg sites and the partial correlation coefficient based on the aging score was calculated based on the normal aged group and each ND group, respectively.
(2) The Benjamin-Hochberg False Discovery Rates (FDR) method was used to adjust the *p*-values of the correlation coefficient as well as the partial correlation coefficient.
(3) The differences of correlation and partial correlation were summarized for each ND group as well as the normal aged group.
(4) The edge between the two cpg sites was retained if the sign of the difference value in the ND group and the normal aged group was opposite, as well as FDR < 0.1 in step (2).
(5) The scale-free characteristics of aging acceleration differential networks were verified by the power-law distribution.
(6) The shortest path between each pair of aging and ND markers was picked out based on each aging acceleration differential network using the Dijkstra algorithm, respectively.
(7) The network was constructed based on the training data and used for further analysis (i.e., identifying the shortest path, exploring potential functions between aging and ND, etc.), and the network constructed based on the test data was used to validate the training network.

As a result, five types of aging differential networks were constructed: AD, PD, PSP, FTD, and all four NDs together.

### Global Sensitivity Analysis Using the Markov Chain Monte Carlo Method

Global sensitivity analysis was used to investigate different cellular homeostasis imbalances in the background of aging acceleration, based on the MCMC method. Both the common and specific characteristics across different ND types were analyzed. To explore the common characteristics among different ND, we made a presumption that if the marker indicated the high risk scores in more than one ND type, then it might reveal the common mechanisms across multiple ND types.

The MCMC method was used for sampling from certain posterior distributions following a given probabilistic background in a high-dimensional space. The key step in MCMC was to construct a Markov chain whose equilibrium distribution equals the target probability distribution. It proceeded as follows:

(1) Construct a transition kernel of an ergodic Markov chain. In this study, the prior distribution for each of the parameters was the normal distribution based on both aging and disease markers for each ND types, respectively.
(1) Simulate the chain until it reaches an equilibrium. The Metropolis-Hastings sampling method was used to determine whether the new sample (θ*^∗^*) is acceptable based on the *α* value:

(6) $$\alpha =\frac{P{\left(\theta^{{}^{*}}|X\right)}^{*}q\left(\theta^{n}\to \theta^{{}^{*}}\right)}{P{\left(\theta^{n}|X\right)}^{*}q\left(\theta^{n}\to \theta^{{}^{*}}\right)}$$

where *P(*θ*^*n*^| X)* and *P(*θ*^∗^| X)* are the posterior probabilities of the n-th accepted sample and the new sample, *q(*θ*^*n*^→*θ*^∗^)* represents the transition probability from the *n* th accepted sample to the new sample, and *q(*θ*^∗^→*θ*^*n*^)* is the transition probability from the new sample to the *n* th accepted sample.

In this article, the mean value of the aging score as well as four types of ND scores were used to evaluate the common characteristics across multiple types of ND. The mean value of the aging score and each ND risk score was used to evaluate the special characteristics of each type of ND.

1. Perform global sensitivity analysis. In this study, the K–S statistic was used to calculate the sensitivity of each parameter.

(7) K-S=sup|F1-F2|

where *F1* was the cumulative distribution of samples with a minus value after normalization, whereas *F2* was the cumulative distribution of samples with the plus value. The interval for the K–S statistic was set to two (based on the sign of normalized DNA methylation profiles). The MCMC pseudocode is provided in Supplementary Text 4, 5.

### Enrichment Analysis

The biological functions of the existing genes were found by enrichment analysis. Gene Ontology (GO) terminology and KEGG pathway were taken from the gene set enrichment analysis (GSEA) platform (version 7.2)^1^. A hypergeometric test was used to estimate the enrichment degree of the KEGG pathway or go BP term. The hypergeometric test formula was:

(8) $$P\left(X\geq x\right)=1-\sum_{k=0}^{x-1}\frac{c_{M}^{k}\times c_{N-M}^{n-k}}{c_{M}^{k}}$$

where *N* was the gene set of the whole gene, *M* was the known genes (e.g., KEGG pathway, or BP terms), *N* was the number of identified genes in each shortest pathway, and *k* was the number of common genes between the known genes and the identified candidate genes (in each aging-ND shortest path). The *p*-values of each pathway were controlled by the Benjamin-Hochberg (BH) method. To ensure the reliability of the results, FDR < 0.05 was selected.

## Data Availability Statement

The original contributions presented in the study are included in the article/Supplementary Material, further inquiries can be directed to the corresponding author/s.

## Author Contributions

FS, YH, and YW performed the algorithm and analyzed the data. FS, YH, YC, XY, and YW wrote the manuscript. XS and YW designed and sponsored the study. All authors read and approved the manuscript.

## Conflict of Interest

The authors declare that the research was conducted in the absence of any commercial or financial relationships that could be construed as a potential conflict of interest.

## Footnotes

1 http://software.broadinstitute.org/gsea/downloads.jsp

## Supplementary Material

Supplementary Figure 1 The results of the Kruskal–Wallis test for age-matched different ND samples and normal samples. **(A)** AD; **(B)** PD; **(C)** PSP; **(D)** FTD.

Supplementary Figure 2 The network marker with the highest betweenness of different ND. **(A)** AD; **(B)** PD; **(C)** PSP; **(D)** FTD.

Supplementary Table 1 The *p*-value of K–S test of the aging score.

Supplementary Table 2 The chronological age and aging scores of each ND group and the normal aged group (after age-matched).

Supplementary Text 1 The detailed datasets used in this work.

Supplementary Text 2 The cpg sites used in this work.

Supplementary Text 3 The selected risk markers of the aging predictor and each ND predictor, along with their ReliefF weights.

Supplementary Text 4 The pseudocode of MCMC for the common characteristics across multiple ND types.

Supplementary Text 5 The pseudocode of MCMC for the special characteristics of each ND type.

## Abbreviations

AD: Alzheimer’s disease
AUC: area Under the ROC Curve
BH: Benjamin-Hochberg
BP: biological process
FDR: false discovery rates
FTD: frontotemporal dementia
GO: Gene Ontology
KEGG: Kyoto Encyclopedia of Genes and Genomes
K–S: Kolmogorov–Smirnov
MCMC: Markov Chain Monte Carlo
ND: neurodegenerative disease
PD: Parkinson’s disease
PSP: progressive supranuclear palsy
ROC: receiver operating characteristic.
