# Supplementary material for: Comparative Analysis of Multiple Neurodegenerative Diseases Based on Advanced Epigenetic Aging Brain
Source: Front Genet. 2021 May 20;12:657636. doi: 10.3389/fgene.2021.657636 (PMC8173158; doi:10.3389/fgene.2021.657636)
Supplement: Supplementary Figure 1 — The results of the Kruskal–Wallis test for age-matched different ND samples and normal samples. (A) AD; (B) PD; (C) PSP; (D) FTD. [file Data_Sheet_1.ZIP › Supplemental files/TableS1-S2.docx]

**Table S1** The p-value of K-S test of the aging score

|  | Score | Score (adjusted by age) |
| --- | --- | --- |
| AD | 3.08186741733943e-35 | 3.30194232415640e-18 |
| PD | 5.07200023169334e-10 | 8.73394152434148e-06 |
| PSP | 2.24907200100323e-38 | 2.96494352458598e-06 |
| FTD | 2.62788142618198e-18 | 0 |
| The whole ND | 4.19704173369191e-91 | 0 |
| Normal aged | 3.51478759640617e-91 | 1.32357877927619e-48 |

**Table S2**  The chronological age and aging scores of each ND group and the normal aged group (after age-matched)

|  | The median  aging score in  control | The median  aging score  in  ND | The mean  aging score  in control | The mean  aging score  in  ND | The  median  age in  control | The  median  age in  ND | The mean  age in  control | The  mean  age in  ND |
| --- | --- | --- | --- | --- | --- | --- | --- | --- |
| AD | 0.895692 | 0.978186 | 0.7858 | 0.863349 | 73 | 79 | 73.70098 | 79.08594 |
| PD | 0.878227 | 0.939411 | 0.763458 | 0.894845 | 67 | 68 | 66.21905 | 70.11111 |
| PSP | 0.887202 | 1.019035 | 0.781013 | 0.985815 | 70 | 70.5 | 69.74412 | 70 |
| FTD | 0.883306 | 0.968045 | 0.774121 | 0.882603 | 68 | 67 | 68.5726 | 66.43089 |
