# Supplementary figures and images for: Comparative Analysis of Multiple Neurodegenerative Diseases Based on Advanced Epigenetic Aging Brain
Source: Front Genet. 2021 May 20;12:657636. doi: 10.3389/fgene.2021.657636 (PMC8173158; doi:10.3389/fgene.2021.657636)

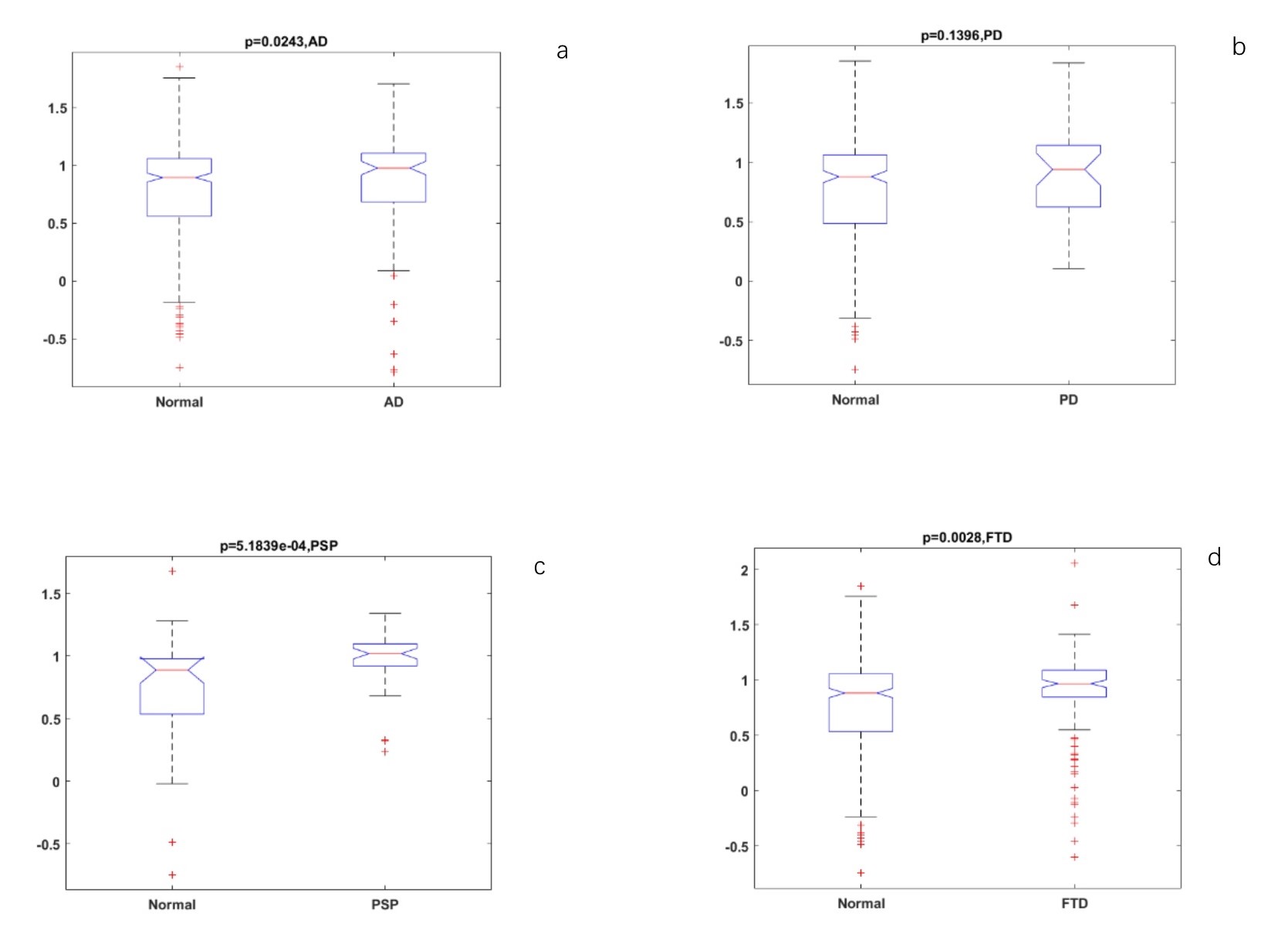

Supplement: Supplementary Figure 1 — The results of the Kruskal–Wallis test for age-matched different ND samples and normal samples. (A) AD; (B) PD; (C) PSP; (D) FTD. [file Data_Sheet_1.ZIP › Supplemental files/Figure S1.tif]

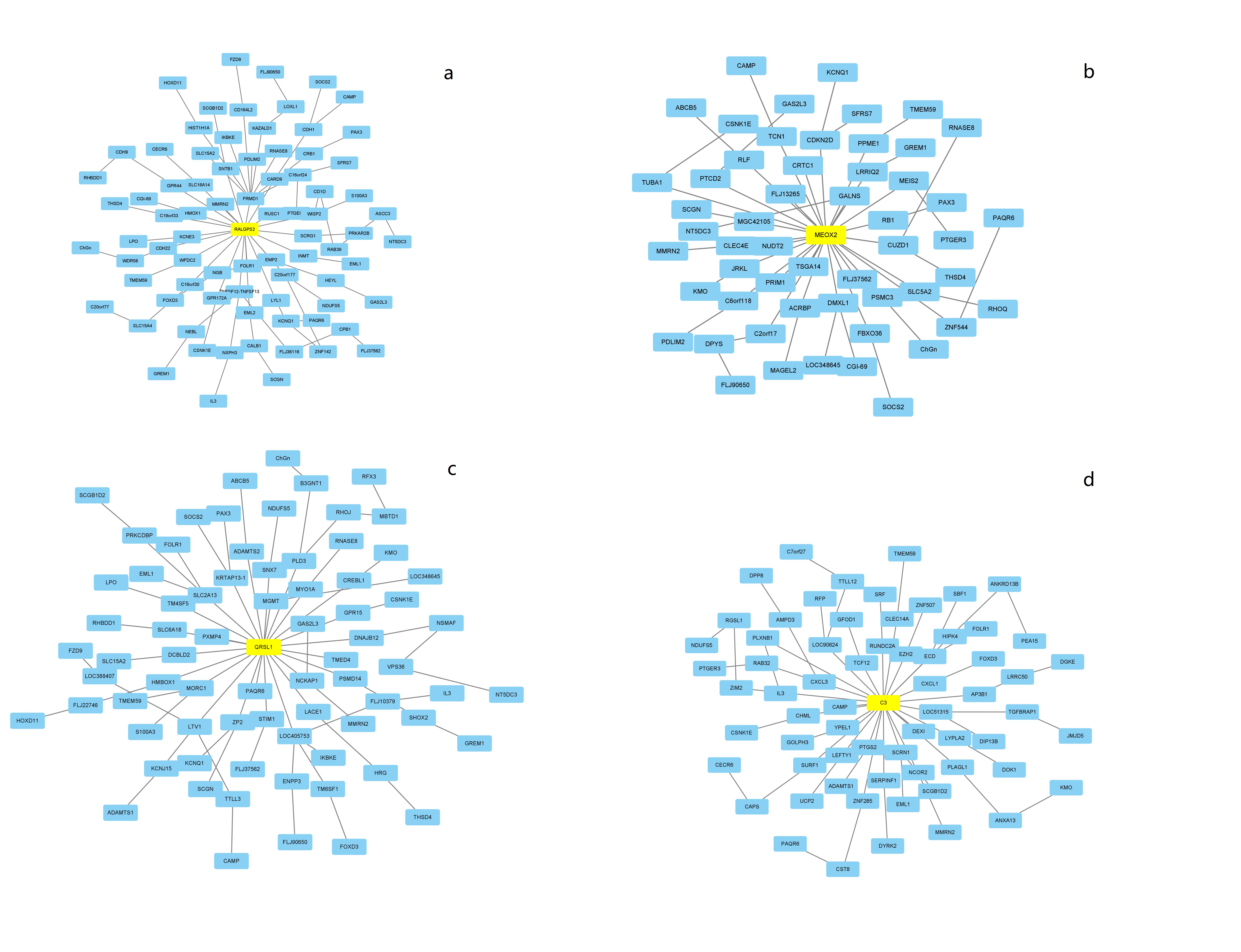

Supplement: Supplementary Figure 1 — The results of the Kruskal–Wallis test for age-matched different ND samples and normal samples. (A) AD; (B) PD; (C) PSP; (D) FTD. [file Data_Sheet_1.ZIP › Supplemental files/FIgure S2.tif]
